# Holocene chloroplast genetic variation of shrubs (*Alnus alnobetula*, *Betula nana*, *Salix* sp.) at the siberian tundra‐taiga ecotone inferred from modern chloroplast genome assembly and sedimentary ancient DNA analyses

**DOI:** 10.1002/ece3.7183

**Published:** 2021-01-31

**Authors:** Stefano Meucci, Luise Schulte, Heike H. Zimmermann, Kathleen R. Stoof‐Leichsenring, Laura Epp, Pernille Bronken Eidesen, Ulrike Herzschuh

**Affiliations:** ^1^ Polar Terrestrial Environmental Systems Research Group Alfred Wegener Institute Helmholtz Centre for Polar and Marine Research Potsdam Germany; ^2^ Institute of Biochemistry and Biology University of Potsdam Potsdam Germany; ^3^ Department of Biology University of Konstanz Konstanz Germany; ^4^ The University Centre in Svalbard Longyearbyen Norway; ^5^ Institute of Environmental Sciences and Geography University of Potsdam Potsdam Germany

**Keywords:** chloroplast genome, genetic variation, hybridization capture, lake sediments, sedimentary ancient DNA (sedaDNA)

## Abstract

Climate warming alters plant composition and population dynamics of arctic ecosystems. In particular, an increase in relative abundance and cover of deciduous shrub species (shrubification) has been recorded. We inferred genetic variation of common shrub species (*Alnus alnobetula*, *Betula nana*, *Salix* sp.) through time. Chloroplast genomes were assembled from modern plants (*n* = 15) from the Siberian forest‐tundra ecotone. Sedimentary ancient DNA (sedaDNA; *n* = 4) was retrieved from a lake on the southern Taymyr Peninsula and analyzed by metagenomics shotgun sequencing and a hybridization capture approach. For *A*. *alnobetula*, analyses of modern DNA showed low intraspecies genetic variability and a clear geographical structure in haplotype distribution. In contrast, *B*. *nana* showed high intraspecies genetic diversity and weak geographical structure. Analyses of sedaDNA revealed a decreasing relative abundance of *Alnus* since 5,400 cal yr BP, whereas *Betula* and *Salix* increased. A comparison between genetic variations identified in modern DNA and sedaDNA showed that *Alnus* variants were maintained over the last 6,700 years in the Taymyr region. In accordance with modern individuals, the variants retrieved from *Betula* and *Salix* sedaDNA showed higher genetic diversity. The success of the hybridization capture in retrieving diverged sequences demonstrates the high potential for future studies of plant biodiversity as well as specific genetic variation on ancient DNA from lake sediments. Overall, our results suggest that shrubification has species‐specific trajectories. The low genetic diversity in *A*. *alnobetula* suggests a local population recruitment and growth response of the already present communities, whereas the higher genetic variability and lack of geographical structure in *B*. *nana* may indicate a recruitment from different populations due to more efficient seed dispersal, increasing the genetic connectivity over long distances.

## Graphical abstract

Climate warming alters plant composition and population dynamics of arctic ecosystems. In particular, an increase in relative abundance and cover of deciduous shrub species (shrubification) has been recorded. We inferred genetic variation of common shrub species (Alnus alnobetula, Betula nana, Salix sp.) through time. Chloroplast genomes were assembled from modern plants (*n* = 15) from the Siberian forest‐tundra ecotone. Sedimentary ancient DNA (sedaDNA; *n* = 4) was retrieved from a lake on the southern Taymyr Peninsula and was analyzed by metagenomics shotgun sequencing and a hybridization capture approach.

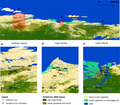

## 1 INTRODUCTION

Ongoing climate change is altering plant composition and biomass in arctic ecosystems. A major contributor to the observed changes is the increase in relative abundance and cover of deciduous shrub species including alder, birch, and willow. This process is colloquially known as shrubification (Crofts et al., 2018; Myers‐Smith et al., 2011). Increased shrub cover alters ecosystem properties. Changes in biodiversity and carbon budgets due to shrubification have been reported, potentially affecting climate warming effects (Blok et al., 2010; Crofts et al., 2018; Lantz et al., 2013; McLaren & Turkington, 2010; Myers‐Smith et al., 2011; Myers‐Smith & Hik, 2013). Species richness, for instance, can decline with increasing shrub dominance across the Arctic (Pajunen et al., 2011). This has been linked to the competitive exclusion of shade‐intolerant species, in particular lichens and mosses growing under shrub canopies (Pajunen et al., 2011; Walker et al., 2006). On the other hand, low densities of shrubs have been associated with increased understory species richness (Crofts et al., 2018).

Drivers of shrubification and its consequences are still poorly understood, particularly with respect to the vast sub‐arctic Siberian areas (Niemeyer et al., 2017). These areas are dominated by alders (*Alnus*), dwarf birches (*Betula*), and willows (*Salix*). For all these taxa, temperature limitation of reproduction most likely determines their current northern extent in the low Arctic (Myers‐Smith et al., 2011), but how they may respond to current changes will probably differ. Shrub species such as *Betula nana* seem to respond fast and tend to outcompete other tundra plants under more favorable growing conditions (e.g., increased air temperatures, higher nitrogen availability; Bret‐Harte et al., 2001, 2002; Myers‐Smith et al., 2011). Alder also shows positive growth responses to increased temperatures; an increase in annual growth of *Alnus fruticosa* was recorded at the southernmost site on Siberia's Yamal Peninsula (Forbes et al., 2010). Shrub willows are also involved in the shrubification of the Russian artic (Myers‐Smith et al., 2011). A strong relationship was found between shrub ring width in an abundant tundra willow and summer temperature for the period 1942–2005 (Forbes et al., 2010). Hence, while a recent increase in shrub biomass is rather well documented, it remains an open question whether the recruitment is a phenological response of the local communities already present or due to recruitment from remote communities.

The expansion or contraction of a species range after changes in biotic or abiotic conditions is the main driver of current genetic structure (Clarke et al., 2019; Epp et al., 2015). Glacial refugia and secondary contact zones are the principal sources of hybridization which increase genetic diversity (Havrdová et al., 2015; Hewitt, 1999; Taberlet et al., 1998), whereas range contraction is most likely to cause loss of genetic diversity, reducing the species’ ability to adapt to a changing climate (Alsos et al., 2012; Vranckx et al., 2012). Decreasing genetic diversity is also associated with increasing distance from refugia (Stamford & Taylor, 2004) as well as with founder effects and bottlenecks following postglacial dispersal (Hewitt, 1996; Jadwiszczak, 2012). However, studies have shown that dispersal ability can counterbalance the loss of genetic diversity due to range contraction in several shrub species (Alsos et al., 2009, 2012).

Changes in shrub abundance and composition along with Siberian treeline changes are well known from the paleo‐record on millennial timescales. *Alnus* sp. is, for instance, known to have retreated southwards during glacial periods and shifted northwards during the late glacial and early Holocene (Klemm et al., 2016; Myers‐Smith et al., 2011; Naito & Cairns, 2011; Zimmermann, Raschke, Epp, Stoof‐Leichsenring, Schirrmeister, et al., 2017), and retreated since then. Continuous vegetation turnover from open forest to single‐tree tundra in southern Taymyr over the last 7,100 years was reflected by a decrease of *Alnus* and the increase of *Salix*, both in a palynological study (Niemeyer et al., 2017) and a metabarcoding study using sedimentary ancient DNA (sedaDNA) records (Epp et al., 2018). These alternating contractions and expansions of shrubs due to climatic fluctuations during the Quaternary have probably affected the geographical distribution of plastid haplotypes in modern shrub populations (Casazza et al., 2016).

Previous studies on modern *Alnus*, *Betula,* and *Salix* have successfully used shotgun metagenomics to explore chloroplast genetic variation and to infer population dynamics (Da̧browska et al., 2006; Eidesen et al., 2015; Jadwiszczak et al., 2012; Salojärvi et al., 2017; Li et al., 2005; Palmé, 2003; Thomson et al., 2015; Wu, 2015; Yang et al., 2018). Locations of glacial refugia and the direction of postglacial migrations of shrubs have been studied in different areas of Europe and Asia (Douda et al., 2014; Gryta et al., 2017; Hantemirova et al., 2018; Havrdová et al., 2015; Mandák et al., 2016; Petit et al., 2003), while the northern Siberian tundra‐taiga ecotone has been largely unexplored.

The chloroplast genome is haploid and maternally inherited in *Alnus*, *Betula,* and *Salix*. It is more prone to stochastic events (i.e., genetic drift) because its effective population size is half that of diploid genomes. In addition, when chloroplast genomes are maternally inherited, the dispersal is limited to seed dispersal that generally occurs over shorter distances than pollen (Lopez et al., 2008). For these two reasons, analyses of genetic variation based on chloroplast genomes usually reveal pronounced geographic structure (Magri, 2008; Petit et al., 1993) that can be suitable for phylogeographic analyses (Gryta et al., 2017).

Since sedaDNA is predominantly of local origin (Parducci et al., 2013, 2015), the comparison of modern DNA from focal species with sedaDNA retrieved from lake sediment cores has great potential to reveal paleovegetation changes in periglacial regions (Jørgensen et al., 2012; Willerslev et al., 2003; Zimmermann, Raschke, Epp, Stoof‐Leichsenring, Schwamborn, et al., 2017). Hitherto, the metabarcoding approach was mainly used to evaluate biodiversity and population dynamics despite its limited information (Epp et al., 2018). Current advances in high‐throughput DNA sequencing technologies allow comprehensive population studies over the whole chloroplast genome, for instance by the combination of shotgun sequencing and hybridization capture. Schulte et al. (2020) show that shotgun sequencing can result in low sequencing coverage of specific taxa of interest due to the extremely high presence of unknown DNA sequences in sedaDNA. To address this problem, they used a hybridization capture approach specifically designed for the *Larix* chloroplast genome. This method successfully enriched targeted sequences and improved accurate detection of genetic variants in sedaDNA (Schulte et al., 2020). However, conserved chloroplast DNA (cpDNA) regions from nontargeted taxa were also captured through the cp conserved regions.

In this study, we compare genetic variation through time to infer shrub population dynamics in common shrub species of the Siberian forest‐tundra ecotone (*Alnus alnobetula, Betula nana, Salix* sp.). We sequenced and assembled 15 chloroplast genomes based on modern DNA (seven *A*. *alnobetula*, seven *B*. *nana,* one *Salix* sp.). The samples of *A*. *alnobetula* and *B. nana* were sampled along a west‐east gradient of the Siberian forest‐tundra ecotone. We identified genetic variations and estimated haplotype networks among the chloroplast genomes of each taxa. To infer historical genetic variation, we used both shotgun and hybridization capture sequencing data retrieved from four sedaDNA samples by Schulte et al. (2020). The samples were obtained from four different age segments of a sediment core from lake CH12 in the Taymyr region. Since the hybridization capture baits were originally designed for the *Larix* chloroplast, only the conserved chloroplast sequences of nontargeted taxa were captured. The sedaDNA sequencing data were taxonomically assigned to *Alnus*, *Betula,* and *Salix* with a lowest common ancestor approach using a chloroplast genome database. Absolute and relative abundances of each of the taxa were identified in order to investigate shrub population dynamics through time. Genetic variations identified in modern individuals were compared with ancient sedaDNA variants. Our study presents new insights of the genomic diversity in *A*. *alnobetula*, *B*. *nana*, and *Salix* sp. in relation to vegetation patterns through space and time.

## 2 MATERIALS AND METHODS

### 2.1 Plant material

**FIGURE 1:**
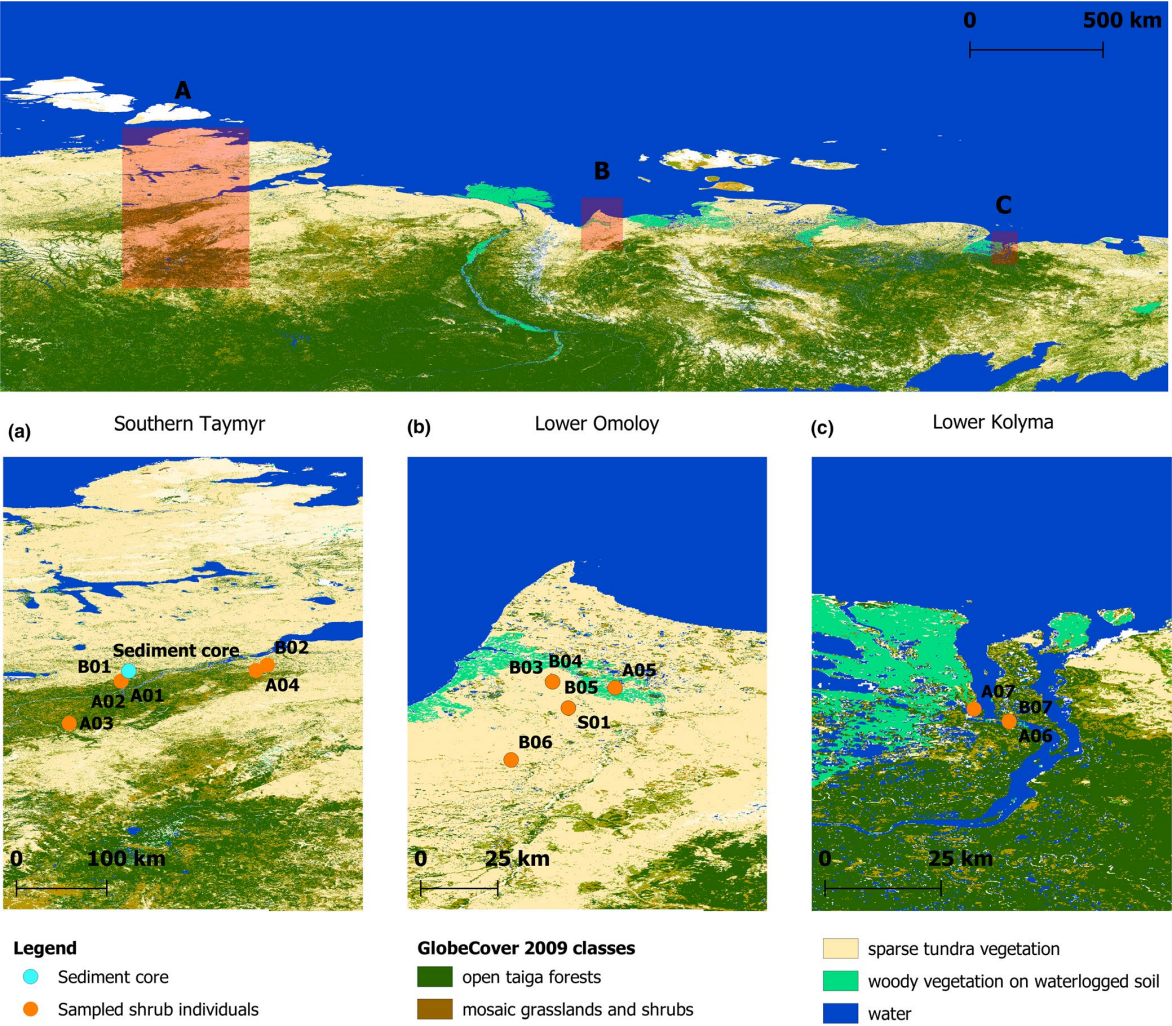
Global composition and land cover map retrieved from the GlobCover Portal (© ESA 2010 and UCLouvain—http://due.esrin.esa.int/page_globcover.php) showing the sampled shrub individuals location as well as the sediment core site at lake CH12

Fresh plant material from seven *Alnus alnobetula* ssp. *fruticosa* (Rupr.) Raus (sample name: A01–A07) and seven *Betula nana* ssp. *exilis* (Sukaczev) Hultén (sample name: B01–B07) samples were collected and identified from three regions located along a west‐east gradient running from the southern Taymyr Peninsula (~97–105°E), the Lower Omoloy River (~132°E), and the Lower Kolyma River (~161°E; Figure 1). In addition, one unknown species of *Salix* sp. (sample name: S01) was collected at the Lower Omoloy River (Table 1). Satellite images and vegetation maps (Stone & Schlesinger, 2003) were used to select uniform vegetation stands within three vegetation types: ‘single‐tree tundra’, ‘forest‐tundra’ (open stands at the northern forest margin), and ‘closed forest’ (Wieczorek et al., 2017). Short twigs with leaves were collected and placed in separate filter bags and dried on silica gel during fieldwork. A reference collection is stored at the Alfred‐Wegener‐Institute Helmholtz Centre for Polar and Marine Research, Potsdam.

**TABLE 1:** List of the sequenced individuals

Sample name	Sample code	Region	Site	Latitude (°N)	Longitude (°E)	Vegetation zone	Species	GenBank accession
A01	SK1386	Taymyr	13TY‐09	72.15067	102.09771	taiga	*Alnus alnobetula* ssp. *fruticosa*	MT872517
A02	SK1388	Taymyr	13TY‐09	72.15067	102.09771	taiga	*Alnus alnobetula* ssp. *fruticosa*	MT872518
A03	SK992	Taymyr	13TY‐07	71.10012	100.81295	taiga	*Alnus alnobetula* ssp. *fruticosa*	MT872519
A04	Beleg 60	Taymyr	13TY‐04‐L10‐09	72.40887	105.44804	taiga	*Alnus alnobetula* ssp. *fruticosa*	MT872520
A05	A2	Omoloy	14OM‐2hS‐1	70.90871	133.07899	polygonal shrub tundra	*Alnus alnobetula* ssp. *fruticosa*	MT872521
A06	Beleg 11	Kolyma	12KO‐04‐II	69.05362	161.205179	taiga	*Alnus alnobetula* ssp. *fruticosa*	MT872522
A07	Beleg 39	Kolyma	12KO‐05	69.11836	161.02342	taiga	*Alnus alnobetula* ssp. *fruticosa*	MT872523
B01	Beleg107	Taymyr	13TY‐09	72.15067	102.09771	taiga	*Betula nana* ssp. *exilis*	MT872524
B02	Beleg19	Taymyr	13TY‐02	72.54772	105.7316	tundra	*Betula nana* ssp. *exilis*	MT872525
B03	B945	Omoloy	14OM‐V3	70.95788	132.57007	tundra	*Betula nana* ssp. *exilis*	MT872526
B04	B922	Omoloy	14OM‐V3	70.95788	132.57007	tundra	*Betula nana* ssp. *exilis*	MT872527
B05	B1	Omoloy	14OM‐V1	70.74418	132.69852	treeline	*Betula nana* ssp. *exilis*	MT872528
B06	B3706	Omoloy	14OM‐2hS‐3	70.32141	132.232316	treeline	*Betula nana* ssp. *exilis*	MT872529
B07	Beleg 7	Kolyma	12KO‐04‐II	69.05362	161.205179	taiga	*Betula nana* ssp. *exilis*	MT872530
S01	S4	Omoloy	14OM‐01‐I	70.74418	132.69852	treeline	*Salix* sp.	MT872531

### 2.2 DNA extraction and sequencing

DNA extraction and sequencing procedure were performed as described in Zimmermann et al. (2019). 20 mg of leaf per individual were transferred into impact‐resistant 2 ml tubes together with two DNA‐free steel beads of 5 mm diameter. The samples were frozen in liquid nitrogen for 3 min and ground to powder with FastPrep‐24 (MP Biomedicals) for 50 s at 4 m/s. The DNeasy Plant Mini Kit (Qiagen) was used to isolate total genomic DNA, according to the manufacturer's protocol with two modifications: (a) 9 µl of 1 M Dithiotreitol (VWR) was added to each sample during lysis after the RNase treatment and (B) the elution was performed twice with 100 µl molecular biology grade water (Omnilab) with an incubation time of 5 min. For each sample, we carried out two extractions (and three extractions for sample B03; Table 1), which were combined and concentrated using the Genomic DNA Clean & Concentrator Kit (Zymo Research Europe GmbH). Gel electrophoresis was performed in order to check the DNA quality, while the DNA concentration was quantified with the Qubit dsDNA BR Assay on the Qubit 2.0 fluorometer (Invitrogen). DNA library preparation, shotgun sequencing, and demultiplexing were performed by the StarSEQ sequencing service. The TruSeq Nano DNA Library Prep Kit (Illumina) was used to build libraries. A distinct index was given to each sample in order to sequence several individuals in parallel (medium size of library = 500 bp). Paired‐end sequencing (2 × 150 bp) was performed on an Illumina NextSeq 500 platform (Illumina).

### 2.3 Sequence processing and de novo assembly of chloroplast genomes

The bioinformatic pipeline followed the procedure described in Zimmermann et al. (2019). The quality check was performed with FastQC (Li et al., 2005). The residual Illumina adapter sequences were then trimmed using Trimmomatic v. 0.3.2 (Bolger et al., 2014; settings: sliding window, window size = 4, average quality = 15, minimum quality to keep a base = 3, minimum length to keep a sequence = 40 nt). De novo assembly of each individual was conducted with CLC Genomics Workbench 8.0 (https://www.qiagenbioinformatics.com/) with an automatic word size, a minimum contig length of 200 nt, and a bubble size of 50. The reads were mapped back to the contigs and the paired‐end information was used to join contigs and build scaffolds. In order to verify the specimen plant identification, the contigs were mapped to all published complete chloroplast genomes of the corresponding genus. The reference chloroplast genomes were downloaded from the National Center for Biotechnology Information Reference Sequence database (NCBI RefSeq; O’Leary et al., 2016).The complete reference chloroplast genomes of *Alnus alnobetula* ssp*. alnobetula* isolate CF09‐AV1 (accession no.: MF136498.1), *Betula nana* ssp. *exilis* (accession no.: KX703002.1) and *Salix purpurea* (accession no.: KP019639) showed the maximum identical sites and pairwise identity. All scaffolds from each sample of the same taxa were mapped to the respective reference chloroplast genome using the function “map to reference” in Geneious Prime (http://www.geneious.com, (Eiter et al., 2003); mapping parameters: maximum gaps per read = 10%, maximum gap size = 50, word length = 24, maximum mismatches = 20%). The aligned scaffolds to the reference chloroplast genomes were overlapping without gaps in all taxa. The consensus was then generated from the multiple alignment in order to obtain the draft chloroplast genome. The Burrows‐Wheeler Aligner v. 0.7.12 (BWA mem default settings; Li & Durbin, 2009) was used to perform the reference‐guided assembly of the trimmed reads of each sequenced individual on the corresponding circular draft chloroplast genome.

### 2.4 Chloroplast genome annotation, variant detection, and haplotype network

The draft chloroplast genomes were annotated integrating cpGAVAS (Liu et al., 2012), Geneious Prime, and tRNAscan‐SE v. 1.21 (Schattner et al., 2005) specifically for transfer RNAs (Lopez et al., 2008; Petit et al., 1993). The circular gene maps were created with OGDraw v1.2 (Lohse et al., 2013). The positions of the start and stop codons of each gene were checked by translating the coding sequences with codon translation table 11. To evaluate genetic variations, a whole genome alignment of all chloroplast genomes of each taxon was conducted with the progressive Mauve v. 2.3.1 (Darling et al., 2010) plugin in Geneious. Single‐nucleotide polymorphisms (SNPs) and insertions and deletions (InDels) among the individual chloroplast genomes were checked through visual inspection. Haplotypes were defined as unique combinations of variants in one individual (Clement et al., 2000). The SNP‐based haplotype networks for the chloroplast (cpDNA) sequences of each taxon were generated by computing absolute pairwise distances and a statistical parsimony approach with TCS (Clement et al., 2000) implemented in PopART v. 1.7 (Population Analysis with Reticulate Trees [Leigh & Bryant, 2015]).

### 2.5 Analyses of sedimentary ancient DNA

Core samples were obtained from a sediment core from lake CH12 (72.399°N, 102.289°E, 60 m a.s.l.) in the Khatanga region of the northern Siberian lowlands, with the Taymyr Peninsula to the north and the Putorana Plateau to the south (Clarke et al., 2019; Wieczorek et al., 2017). The sampling location is in the northern part of the treeline ecotone and currently surrounded by a vegetation of single‐tree tundra. The lake catchment is mainly characterized by southern shrub tundra dominated by Ericaceae dwarf‐shrubs. *Alnus alnobetula* and *B*. *nana* have a scattered distribution and low stature. *Salix* ssp. grows predominantly along the river and lake shorelines (Klemm et al., 2016).

Laboratory work and data processing were performed by Schulte et al. (2020). Core samples were collected at the following depths/ages: 121.5 cm/~6,700 cal yr BP, 87.5 cm/~5,400 cal yr BP, 46.5 cm/~1900 cal yr BP, and 2.5 cm/~60 cal yr BP. The four samples were sequenced with both hybridization capture and shotgun sequencing methods. Hybridization capture baits were specifically designed by Zimmermann et al. (2019) for targeting the *Larix gmelinii* (NCBI accession no.: MK468637.1 [Zimmermann et al., 2019]) chloroplast genome. As parts of the chloroplast genome are highly conserved across taxa (Green, 2011), plant chloroplast DNA of nontargeted taxa were captured. The sequenced reads of the capture and shotgun datasets were taxonomically classified at the genus level using both Ganon IV (Piro et al., 2020) and Kraken 2 v.2.0.7‐beta (Wood et al., 2019) with default parameters against a custom database of 8,132 complete plant chloroplast genomes (downloaded from NCBI in July 2019). Reads classified as one of the shrub taxa of interest (*Alnus*, *Betula*, or *Salix*) were extracted and used for further analyses. For each taxon‐specific subset of sequences, the reads were mapped against the corresponding draft chloroplast genome using BWA aln algorithm (v. 0.7.12; Li & Durbin, 2009). To confirm the reads sequence identity with the species of modern individuals, 100 randomly picked reads were verified with Blast (Altschul et al., 1990). Assembled and unassembled reads were processed separately. PCR duplicates were removed with samtools markdup (Li et al., 2009). Ancient DNA damages were estimated using mapDamage (v. 2.0.8; Jónsson et al., 2013) by Schulte et al. (2020), in order to prove the authenticity of the obtained data.

To compare genetic variations of modern individual with sedaDNA variants, a whole genome alignment of all the individuals’ chloroplast genomes with all capture and shotgun classified reads for each corresponding taxon was computed with Geneious Prime. The variants of capture and shotgun reads were checked through visual inspection. In order to evaluate the hybridization efficiency, the percentage of pairwise identity between *L. gmelinii* (NCBI accession no.: MK468637.1) and *A. alnobetula*, *B. nana,* and *Salix* sp. draft chloroplast genomes was calculated through the function “distance ‐ % of bases/residues which are identical” of the plugin MAFFT alignment v7.450 (Katoh, 2002; Katoh & Standley, 2013). Due to the overall low depth of coverage and partial breadth of coverage, genetic variation within sedaDNA could not be accurately evaluated. To substantiate the reliability of sedaDNA, all capture and shotgun classified reads were aligned separately to each *Alnus* individuals’ chloroplast genomes using the function “map to reference” on Geneious Prime (mapping parameters: maximum gaps per read = 5%, maximum gap size = 50, word length = 24, maximum mismatches = 5%) in order to evaluate the coverage and the number of identical sites.

## 3 RESULTS

### 3.1 Chloroplast genome assembly and genetic variation of modern individuals

#### 3.1.1 Chloroplast genome structure of *A. alnobetula*, *B. nana,* and *Salix* sp. individuals

The *A. alnobetula* draft chloroplast genome was generated from 66,582,099 paired reads that passed trimming. *Betula nana* and *Salix* sp. draft chloroplast genomes were assembled from 55,566,536 and 7,400,002 paired reads, respectively. Maximum contig lengths obtained by the individual assemblies range between 47,614 and 89,456 bp in *A. alnobetula*; 67,592 to 124,641 bp in *B. nana,* and 84,703 bp in *Salix* sp.

The *A. alnobetula* complete chloroplast genomes from 7 individuals ranged from 160,479 to 160,502 bp in length. The complete chloroplast genomes from 7 individuals of *B. nana* showed a wider range of length (160,343 to 160,525 bp), mostly due to a 70 bp InDel among the *B. nana* individuals (Table S1). To account for this variation, two different draft chloroplast genomes were used for *B. nana*. To assemble the unknown *Salix* sp. (S01), all contigs were mapped to each *Salix* reference chloroplast genome available. *Salix purpurea* (accession no.: KP019639) showed the most identical sites and highest pairwise identity and was selected as the reference chloroplast genome. The size of the single assembled *Salix* sp. chloroplast genome was 155,784 bp.

The minimum depth of coverage per base among all individual chloroplast genomes varied between 16x (B07) and 991x (B04). All chloroplast genomes showed a typical quadripartite structure that consists of a pair of inverted repeat (IR) regions, separated by the long single copy (LSC) and short single copy (SSC) regions. The length of the IR varied from 26,031 bp in *A. alnobetula*, 25,865 bp in *B. nana* to 27,459 bp in *Salix* sp. The GC content was 36.4%, 36.1%, and 36.7% for *A. alnobetula*, *B. nana,* and *Salix* sp. respectively.

**FIGURE 2:**
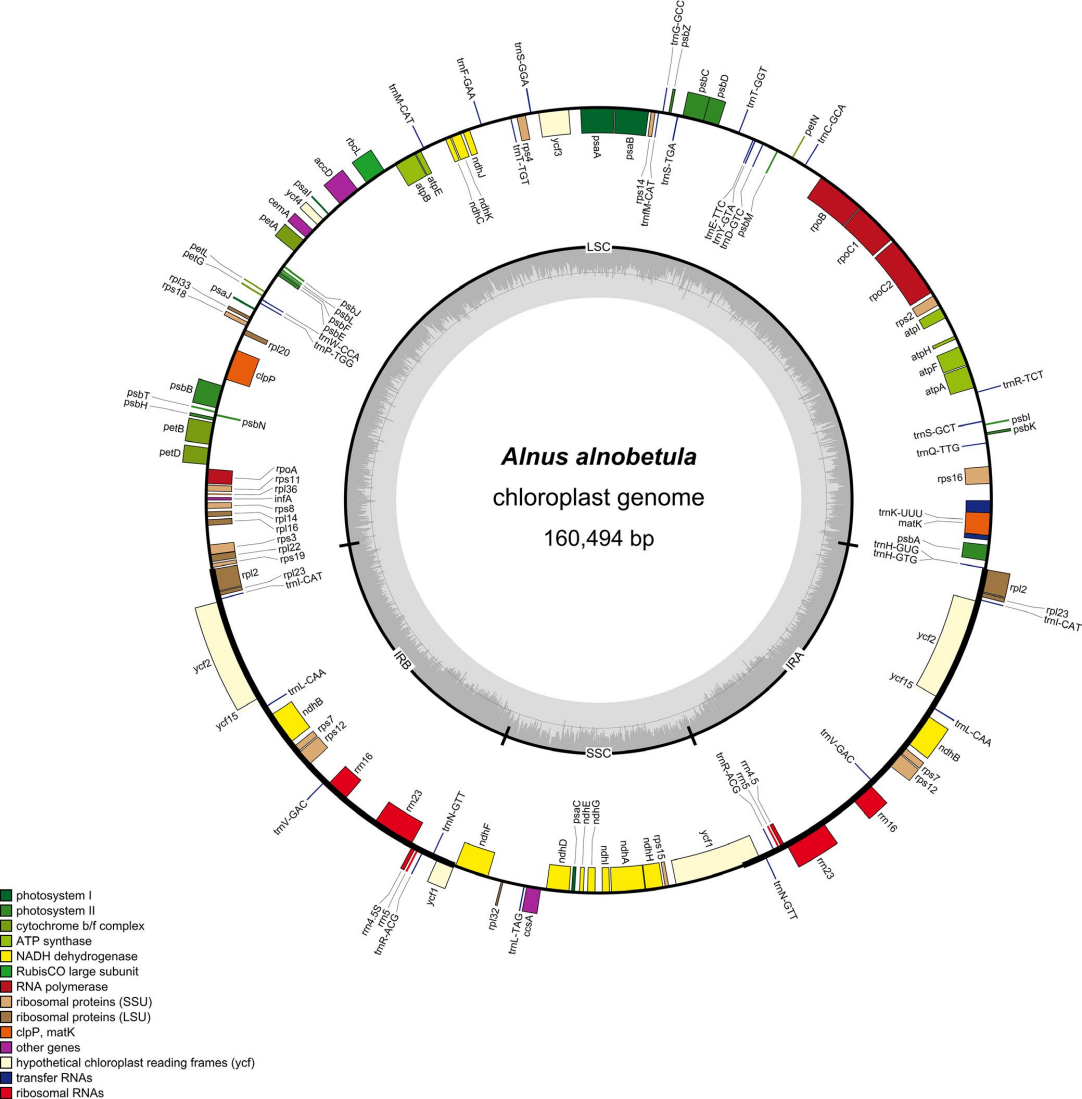
Circular gene map of *A. alnobetula* draft chloroplast genomes. Genes outside the circle are transcribed clockwise while genes inside the circle are transcribed counter‐clockwise

The annotated draft chloroplast genome obtained from the seven *A. alnobetula* individuals had a length of 160,494 bp. The chloroplast genome had 123 genes, including 85 protein‐coding genes, 30 tRNA genes, and 8 rRNA genes (Figure 2). In the two IR regions, six protein‐coding genes (ndhB, rpl23, rps7, rps12, ycf2, ycf15), four rRNA genes, and five tRNA genes were duplicated. All chloroplast genomes had the same gene number and gene order as observed in the *Alnus alnobetula* (accession no.: MF136498.1) reference chloroplast genome. An extension of 29 bp at the IRb/SSC border and a contraction of 7bp at the LSC/IRb border were present in all individuals in contrast to the reference.

**FIGURE 3:**
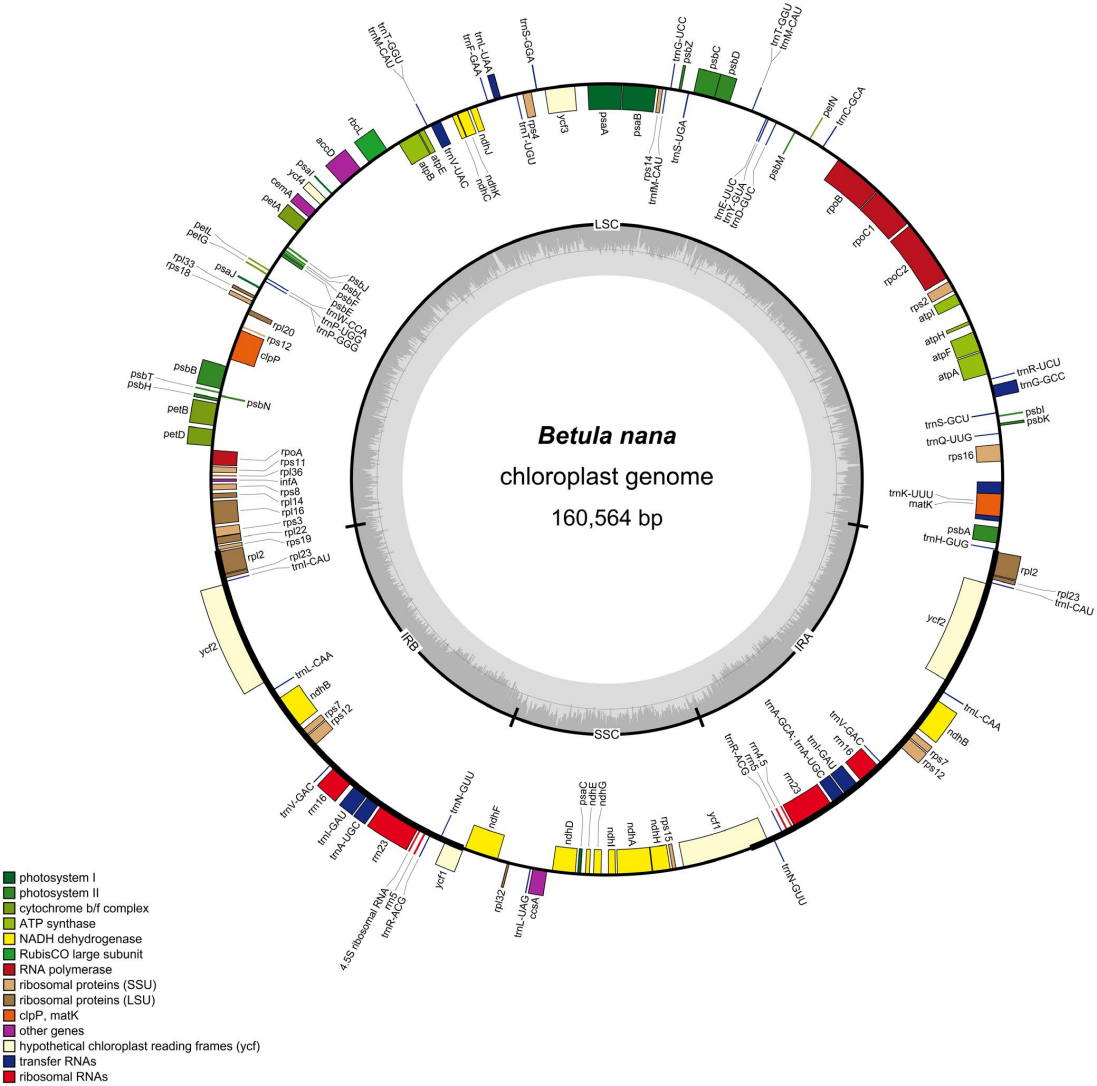
Circular gene map of *B. nana* draft chloroplast genomes. Genes outside the circle are transcribed clockwise while genes inside the circle are transcribed counter‐clockwise

The annotated draft chloroplast genome obtained from the *7 B. nana* individuals had a length of 160,564 bp. The chloroplast genome had 134 genes, including 86 protein‐coding genes, 8 ribosomal RNA genes, and 40 tRNA genes (Figure 3). Twenty genes occurred as double copies in the IR regions, including all rRNA species (4.5S, 5S, 16S, 23S rRNA), 9 tRNA (trnA‐UGC, trnI‐CAU, trnI‐GAU, trnL‐CAA. trnM‐CAU, trnN‐GUU, trnR‐ACG, trnT‐GGU, trnV‐GAC), and 7 protein‐coding genes (ndhB, rpl2, rpl23, rps7, rps12, ycf1, ycf2; Hu et al., 2017). All chloroplast genomes had the same gene number and gene order as observed in the *B. nana* (accession no.: KX703002.1) reference chloroplast genome. The locally collinear blocks (LCBs) identified by the Mauve aligner reveal high sequence similarity (~99%) across the IR regions between *A. alnobetula* and *B*. *nana*, while the SSC and LSC regions show ~85% and ~75% pairwise identity, respectively.

**FIGURE 4:**
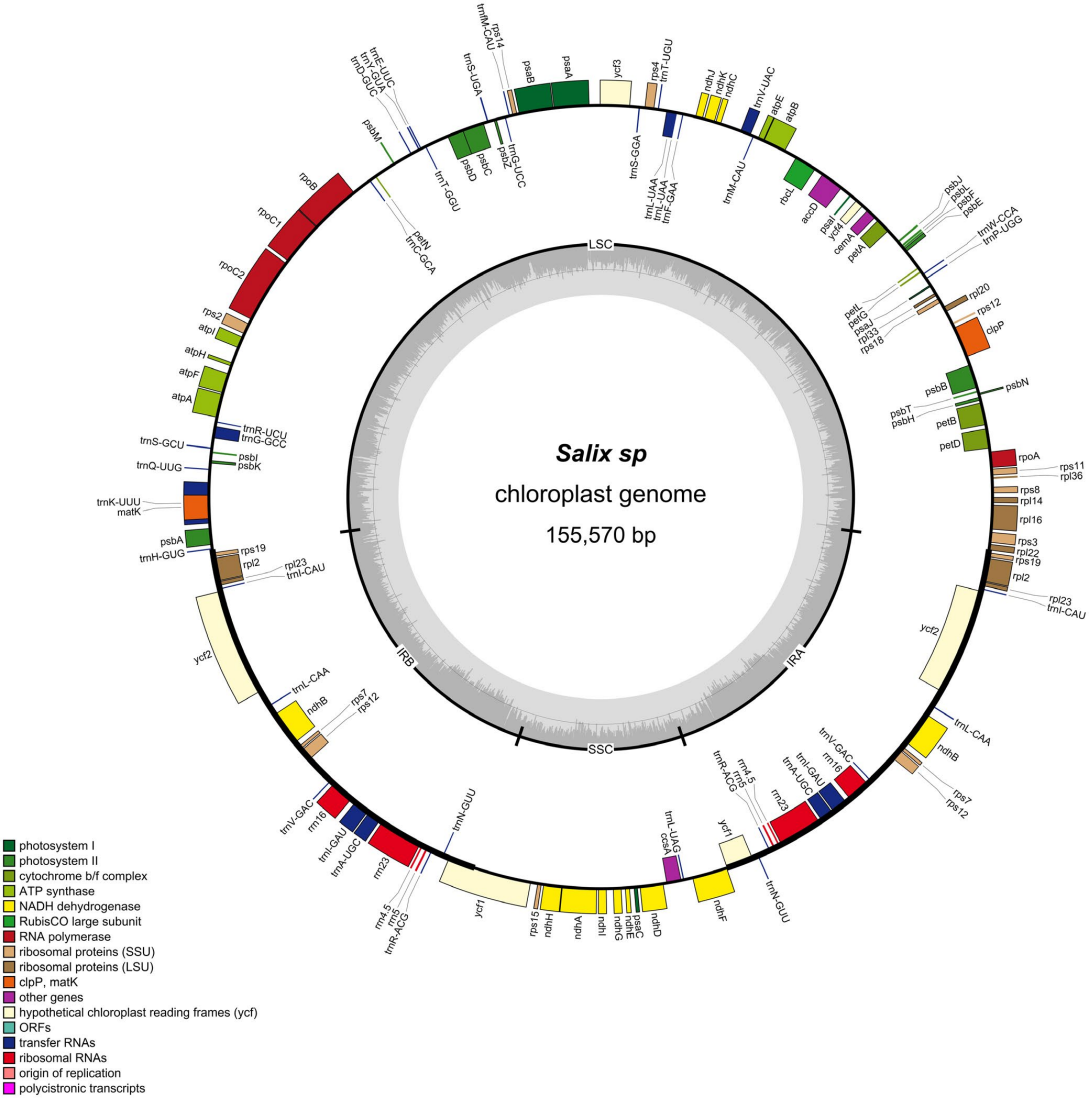
Circular gene map of *Salix* sp. draft chloroplast genomes. Genes outside the circle are transcribed clockwise while genes inside the circle are transcribed counter‐clockwise

The chloroplast genome of the *Salix* sp. individual had a length of 155,570 bp and included 110 unique genes, whereof 76 protein‐coding genes, 30 tRNA genes, and four rRNA genes (Figure 4). This matched the *S. purpurea* chloroplast genome used as a reference. A total of 18 of these genes (seven tRNAs, seven protein genes, four rRNAs) were duplicated in the IR regions. The genes rpl22 and ycf1 span the boundary of IRb/LSC and IRa/SSC, respectively, which were partially duplicated in the IR regions forming pseudogenes. The rps12, which encodes the 30S ribosomal protein S12, was a trans‐spliced gene with the 5′‐end located in the LSC region and the duplicated 3′‐end located in the IR regions. The trnK‐UUU had the largest intron (2,552 bp), which contains the matK gene.

#### 3.1.2 Genetic variation and haplotype network

**FIGURE 5:**
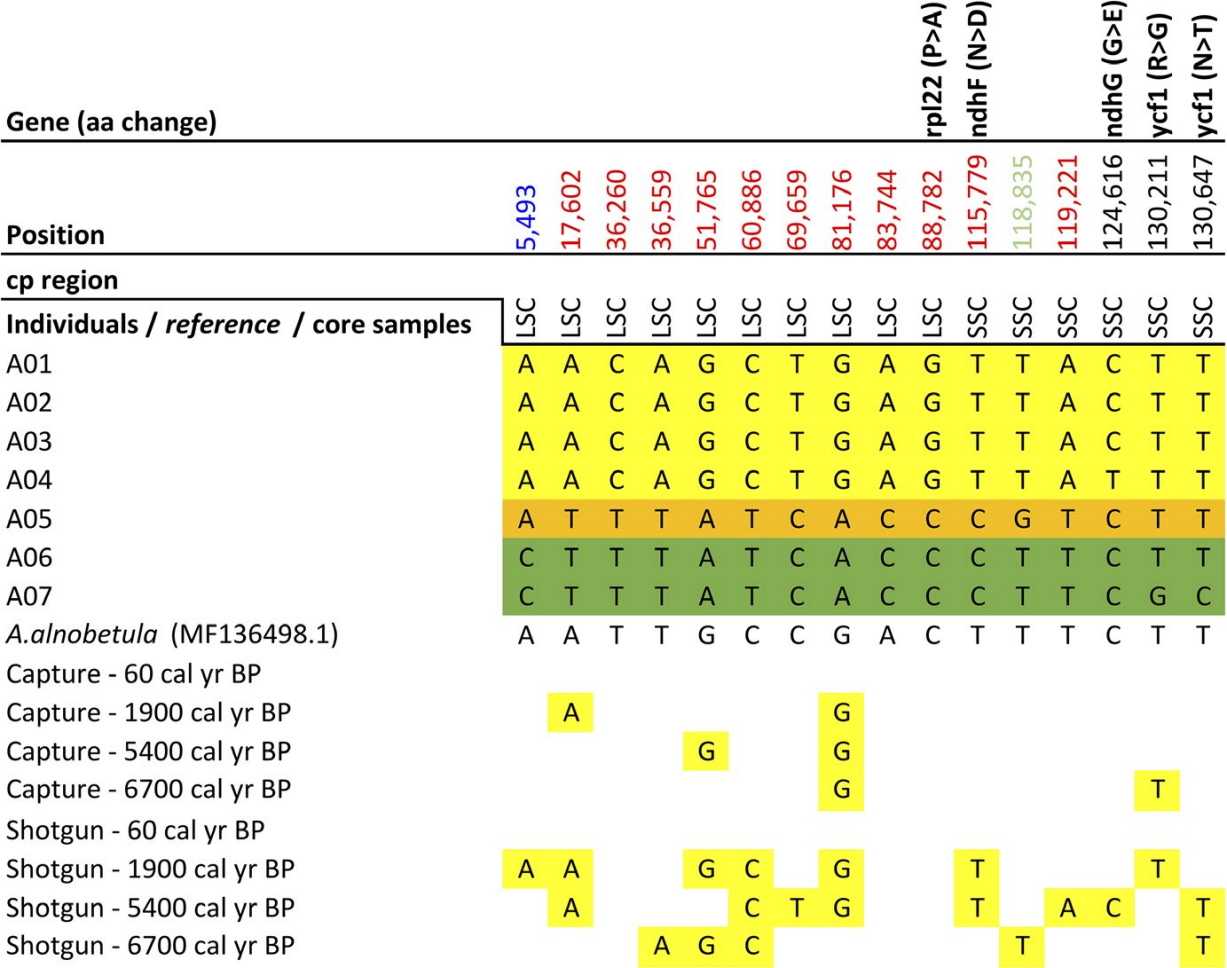
Single‐nucleotide polymorphisms (SNPs) detected in the whole chloroplast gnome alignment of the seven *Alnus alnobetula* individuals. The reference chloroplast genome and the sedaDNA retrieved from core samples through hybridization capture and shotgun sequencing methods were mapped subsequently in order to evaluate their variants corresponding to the SNPs’ positions. An SNP’s position corresponds to the reference chloroplast genome. If an SNP is located within a gene, the corresponding gene name is given in the first row. If no reads were retrieved from core samples, no variant is reported. Color code: Taymyr specific variation = yellow; Omoloy specific variation = orange; Kolyma specific variation = green; potential marker for Taymyr geographic discrimination = position highlighted in red; potential marker for Kolyma geographic discrimination = position highlighted in blue; potential marker for Omoloy geographic discrimination = position highlighted in light green

The complete chloroplast genome alignment of *Alnus* revealed 16 SNPs (eight transversions, eight transitions; Figure 5), five InDels (Table S2), and 13 homopolymer stretch differences (4× poly(T), 8× poly(A), 1× poly(G)). Five SNPs located in coding regions led to change of the translated amino acid (aa) sequence. The SNPs on *ndhF, ndhG,* and *ycf1* were singletons and the amino acid property also changed (Table 2). One SNP on the *ndhF*, *ndhG*, and *rpl22* genes was shared by the individuals from Omoloy (A05) and Kolyma (A06, A07). Two SNPs on *ycf1* were only present in one out of two individuals from Kolyma (A07). *ycf1* and *ndhF* were located on either side of the junctions IRb/SSC/Ira. In all seven chloroplast genomes, the *ycf1* gene crossed the SSC/IRa boundary region, resulting in the incomplete duplication of this gene in two IRs. The *rpl22* gene was also close to the LSC/IRb junction. In noncoding regions, there were 11 SNPs, of which one was a singleton.

**TABLE 2:** Chloroplast genes with nonsynonymous single‐nucleotide polymorphisms (SNPs), the SNP position on the respective reference chloroplast genome, and the changes in amino acid properties

Taxa	Genes	Chloroplast region	SNP position	Amino acid change	Amino acid property changes
*A. alnobetula*	*rpl22*	LSC	88,782	Proline (P) > Alanine (A)	Hydrophile > hydrophile
*A. alnobetula*	*ndhF*	SSC	115,779	Asparagine (N) > Aspartic acid (D)	Hydrophile > Acid
*A. alnobetula*	*ndhG*	SSC	124,616	Glycine (G) > Glutamic acid (E)	Hydrophile > Acid
*A. alnobetula*	*ycf1*	SSC	130,211	Arginine (R) > Glycine (G)	Basic > Hydrophile
*A. alnobetula*	*ycf1*	SSC	130,647	Asparagine (N) > Threonine (T)	Hydrophile > hydrophile
*B. nana*	*psaB*	LSC	1,475	Glycine (G) > Serine (S)	Hydrophile > hydrophile
*B. nana*	*petD*	LSC	82,030	Aspartic acid (D) > Alanine (A)	Acid > Hydrophile
*B. nana*	*rps8*	LSC	85,230	Arginine (R) > Glutamine (Q)	Basic > Hydrophile
*B. nana*	*ccsA*	SSC	120,504	Leucine (L) > Phenylalanine (F)	Hydrophope > Aromatic
*Salix* sp.	*rpoB*	LSC	132,213	Valine (V) > Isoleucine (I)	Hydrophope > Hydrophope
*Salix* sp.	*rpoC1*	LSC	135,800	Threonine (T) > Methionine (M)	Hydrophile > Hydrophope

Eleven SNPs showed one variant among the individuals from Taymyr and another variant among the individuals from both Omoloy and Kolyma (Figure 5, position highlighted in red), displaying potential as spatially diagnostic markers for the Taymyr region. One variation (Figure 5, position highlighted in light green) was a potential marker for Omoloy geographic discrimination and another variation for Kolyma (Figure 5, position highlighted in blue). Considering both SNPs and InDels, the number of pairwise differences between the chloroplast genomes ranged from a minimum of three among the individuals from the Taymyr region to a maximum of 13 between the individuals from Taymyr and Kolyma/Omoloy regions.

**FIGURE 6:**
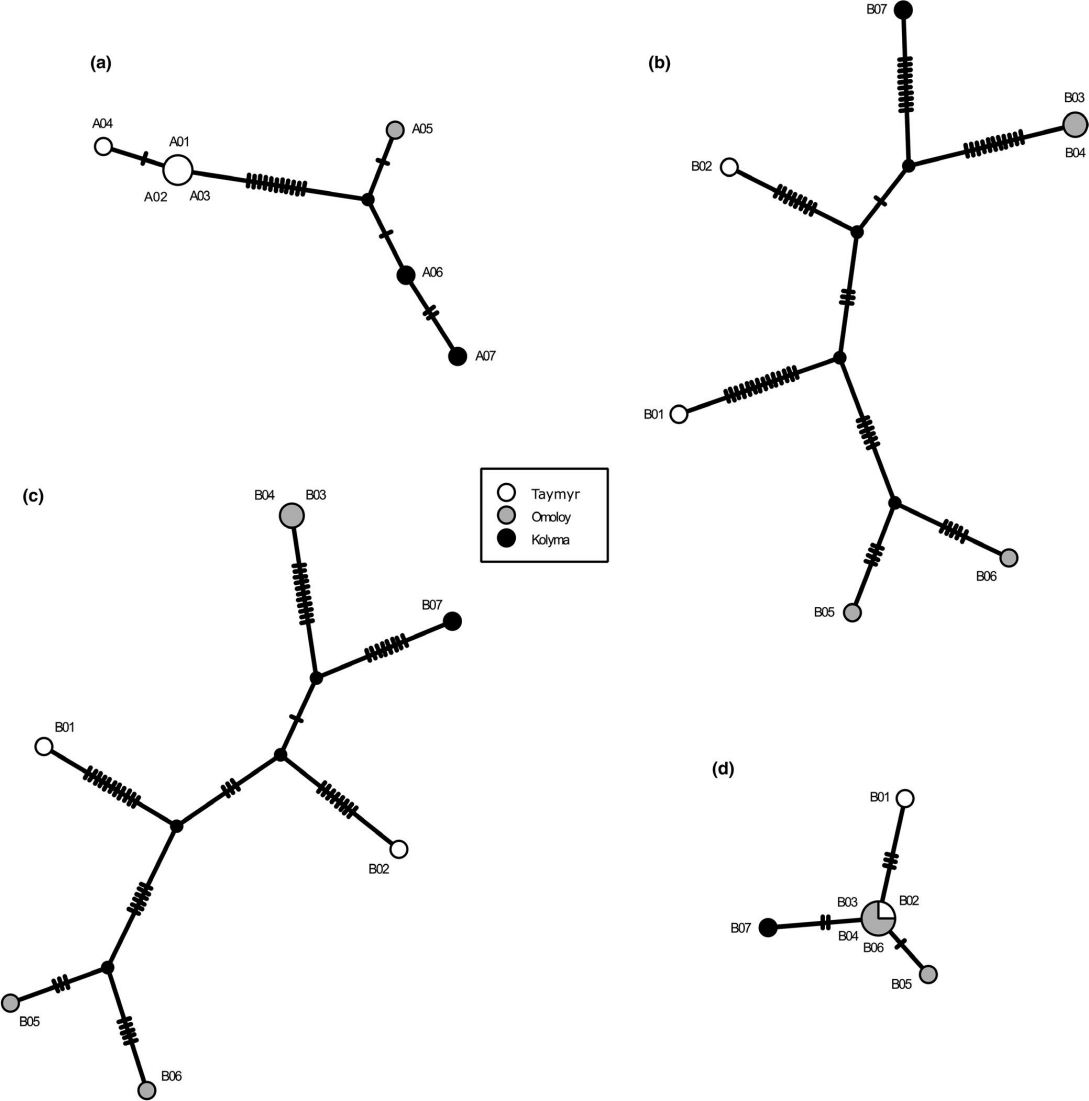
Statistical parsimony haplotype networks for (a) all chloroplast single‐nucleotide polymorphisms (SNPs) in *Alnus* individuals, (b) all SNPs in *Betula* individuals, (c) SNPs in noncoding regions in *Betula* individuals, and (d) SNPs within genes in *Betula* individuals, with haplotypes as circles. The sizes of the circles are proportional to the number of individuals exhibiting this haplotype and are colored according to their region of origin. Small black circles represent estimated putative intermediate haplotypes. Hatch marks along the branches indicate mutations between the nodes

The TCS haplotype network based on the 16 previously described SNPs from *A. alnobetula* individuals showed a clear spatial signal between the haplotypes from Taymyr, Omoloy, and Kolyma regions (Figure 6a). Haplotypes within the Omoloy and Kolyma populations appeared closest together in the haplotype network.

**FIGURE 7:**
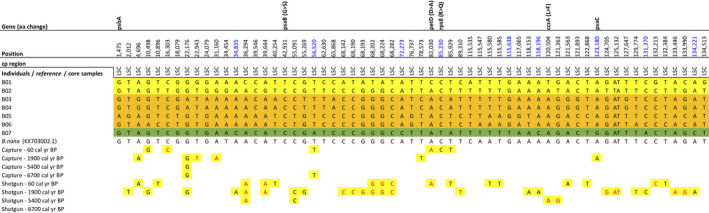
Single‐nucleotide polymorphisms (SNPs) detected in the whole chloroplast gnome alignment of the seven *B. nana* individuals. The reference chloroplast genome and the sedaDNA retrieved from core samples through hybridization capture and shotgun sequencing methods were mapped subsequently in order to evaluate their variants corresponding to the SNPs’ positions. An SNP’s position corresponds to the reference chloroplast genome. If an SNP is located within a gene the corresponding gene name is given in the first row. If no reads were retrieved from core samples, no variant is reported. Color code: Taymyr specific variation = yellow; Omoloy specific variation = orange; Kolyma specific variation = green; Potential marker for Kolyma geographic discrimination = position highlighted in blue. The core sample's variants highlighted in red match with only one out of two individuals from Taymyr

The complete chloroplast genome alignment of 7 *B. nana* individuals revealed 61 SNPs (Figure 7), 15 InDels (Table S2), and 55 homopolymer stretch differences (30x poly(T), 24x poly(A), 1x poly(G)). There were six singleton SNPs located in the coding regions, four of which led to a change in the translated amino acid sequence and its properties (Table 2). Nine variations, two of which were located on the genes *rps8* and *psaC,* showed one variant only present on the individual from Kolyma while another variant was present in the individuals from Taymyr and Omoloy (Figure 7 – position highlighted in blue). There were no variations shared by all individuals from Omoloy or Kolyma. In noncoding regions, we detected 55 SNPs, of which 33 were singletons. A 70 bp deletion occurred in the noncoding region *rpl22‐rps19* of one individual from Taymyr (B01) and two individuals from Omoloy (B05, B06). We detected 25 pairwise differences between the chloroplast genomes of the two individuals from the Taymyr region and 30 pairwise differences between the individuals from Omoloy.

No clear spatial signal was detected among the *B. nana* haplotypes. The Omoloy haplotypes form two distinctive groups, with the Taymyr and Kolyma haplotypes between them (Figure 6b). The haplotype network was repeated for SNPs only in noncoding regions and for SNPs only in coding regions (Figure 6c–d). The haplotype network showed a stronger spatial signal for SNPs in coding regions.

**FIGURE 8:**
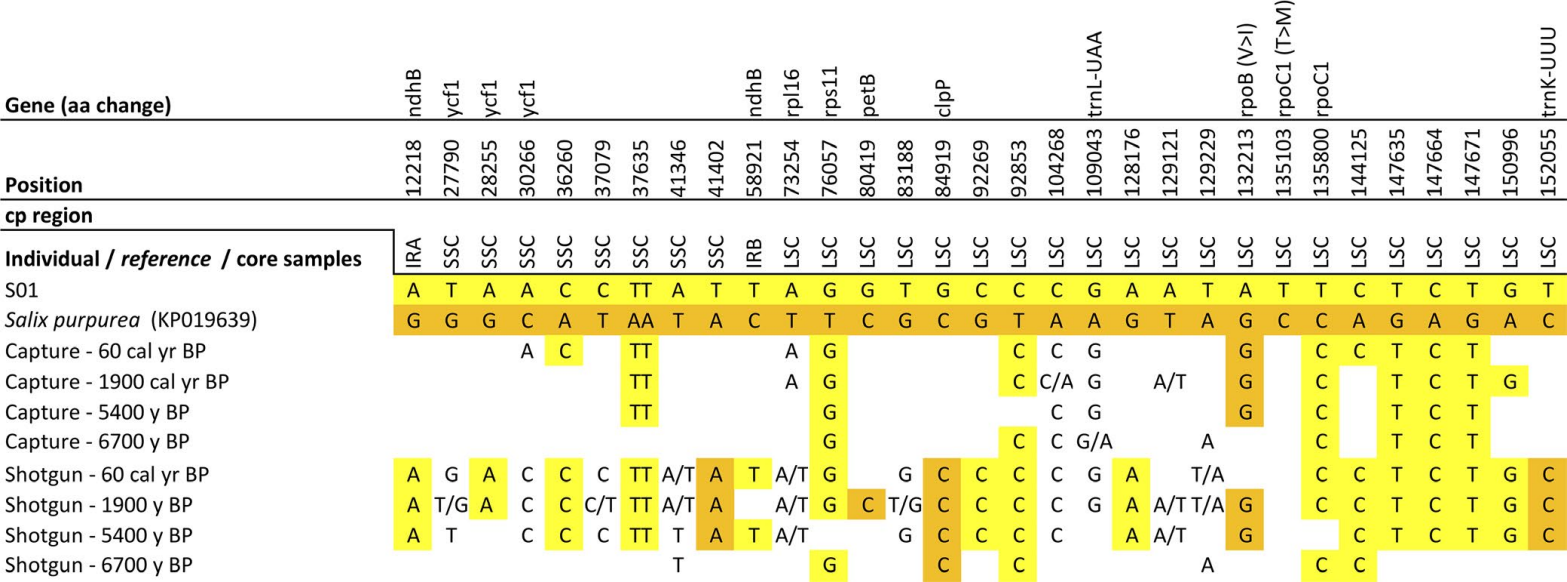
Single‐nucleotide polymorphisms (SNPs) detected in the whole chloroplast gnome alignment between a *Salix* sp. individual (S01) and the reference *Salix purpurea* (accession no.: KP019639). The reference chloroplast genome and the sedaDNA retrieved from core samples through hybridization capture and shotgun sequencing methods were mapped subsequently in order to evaluate their variants corresponding to the SNPs’ positions. An SNP’s position corresponds to the reference chloroplast genome. If an SNP is located within a gene, the corresponding gene name is given in the first row. If no reads were retrieved from core samples, no variant is reported. Color code: variations corresponding exclusively to the individual S01 = yellow; variations corresponding exclusively to *Salix purpurea* = orange

The whole chloroplast genome alignment between the *Salix* individual S01 (from Omoloy) and the reference *Salix purpurea* (accession no.: KP019639) had 31 SNPs (Figure 8), 9 InDels (Table S2), and 55 homopolymer stretch differences (24x poly(T), 31x poly(A), 1x poly(C)). Transversions made up 58% (Vranckx et al., 2012) of the SNPs, while 42% (Epp et al., 2015) of the SNPs are transitions. 14 SNPs were located within genes, of which five were located in introns. Two SNPs led to changes in the translated amino acid sequence. In one case, the amino acid property also changed (Table 2). One SNP was located on *ndhB,* which was doubled in the IRs regions. Three SNPs were located on *ycf1*, which crossed the IRb/SSC boundary region, resulting in the incomplete duplication of this gene in two IRs. Furthermore, two SNPs were detected in transfer RNA genes, *trnL‐UAA,* and *trnK‐UUU*. Two InDels were in intragenic regions of *rpl16* and *clpP* genes.

### 3.2 Chloroplast genetic variation in lake sediment core samples from Taymyr

#### 3.2.1 Taxonomic assignment, reads counts, and breadth of coverage

**FIGURE 9:**
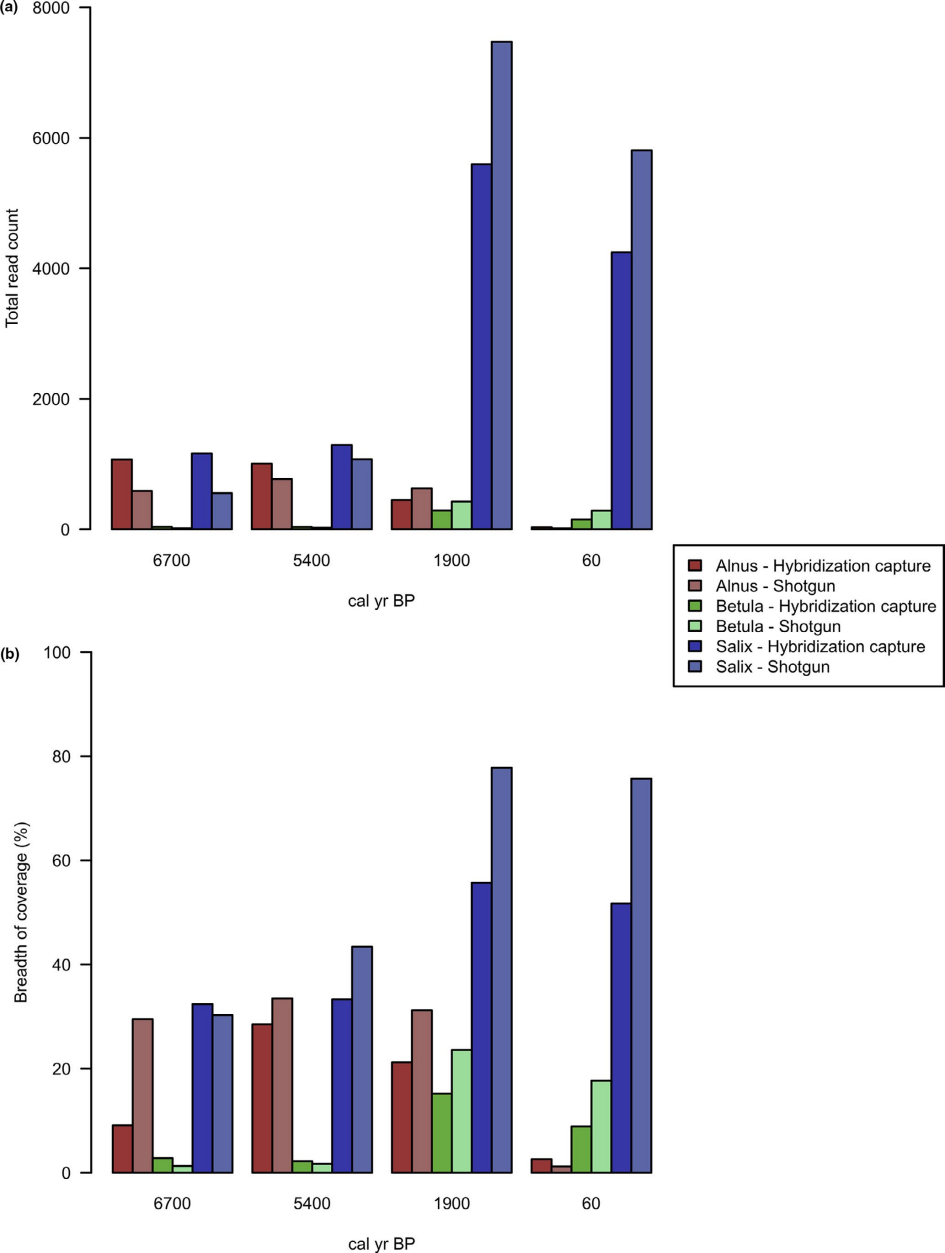
(a) Total read count and (b) breadth of coverage of hybridization capture and shotgun datasets

Only the conserved chloroplast regions between *L. gmelinii* and *Alnus*, *Betula,* and *Salix* were captured since the hybridization capture baits were specifically designed for targeting *L. gmelinii* (Schulte et al., 2020; Zimmermann et al., 2019). The percentage of pairwise identity between the chloroplast genomes of *L. gmelinii* and *A. alnobetula*, *B. nana,* and *Salix* sp. was 28.1%, 32.2%, and 38.3%, respectively. Taxonomic assignment was performed in order to extract the reads classified as *Betula*, *Alnus*, or *Salix* using both Ganon IV (Piro et al., 2020) and Kraken 2 v.2.0.7‐beta (Wood et al., 2019) tools. Ganon IV (Table 3) showed 10%–40% higher assigned read counts depending on the sample, in both hybridization capture and shotgun datasets. However, the proportion of read counts among the samples was comparable with the results obtained by Kraken 2 (Table S3). The hybridization capture data had a lower breadth of coverage then the metagenomics shotgun data on average (Table 3). Despite this, the ratio of read counts and breadth of coverage was proportional and comparable between the hybridization capture and shotgun data (Table 3; Figure 9). Overall, *Salix* and *Betula* chloroplast genomes had the highest and the lowest breadth of coverage, respectively. Hybridization capture sequencing data had a higher rate of PCR duplicates due to additional 18 amplification cycles performed after capturing.

**TABLE 3:** Taxonomic assignment of reads to *Alnus*, *Betula,* and *Salix* for each capture and shotgun sample, performed with Ganon IV (Materials and methods)

Dataset	Assembled reads (+duplicates)	Unassembled reads (+duplicates)	Total read counts	Breadth of coverage (%)	Relative (%)
Alnus—Capture—60 cal yr BP	32 (1,421)	1 (5)	33	2.60	0.74
Alnus—Capture—1900 cal yr BP	409 (43,751)	41 (1,053)	450	21.20	7.11
Alnus—Capture—5,400 cal yr BP	953 (46,091)	53 (660)	1,006	28.50	43.08
Alnus—Capture—6,700 cal yr BP	1,008 (43,919)	64 (623)	1,072	29.10	47.16
Alnus—Shotgun—60 cal yr BP	16 (22)	0	16	1.20	0.26
Alnus—Shotgun—1900 cal yr BP	609 (721)	18 (20)	627	31.20	7.35
Alnus—Shotgun—5,400 cal yr BP	763 (808)	9 (11)	772	33.50	41.20
Alnus—Shotgun—6,700 cal yr BP	578 (617)	9 (10)	587	29.50	50.60
Betula—Capture—60 cal yr BP	137 (18,703)	15 (449)	152	8.90	3.43
Betula—Capture—1900 cal yr BP	259 (26,067)	29 (985)	288	15.20	4.55
Betula—Capture—5,400 cal yr BP	33 (1,593)	3 (80)	36	2.20	1.54
Betula—Capture—6,700 cal yr BP	35 (1,189)	2 (7)	37	2.80	1.63
Betula—Shotgun—60 cal yr BP	281 (347)	5 (5)	286	17.70	4.68
Betula—Shotgun—1900 cal yr BP	412 (483)	13 (13)	425	23.60	4.98
Betula—Shotgun—5,400 cal yr BP	27 (29)	0	27	1.70	1.44
Betula—Shotgun—6,700 cal yr BP	18 (19)	0	18	1.30	1.55
Salix—Capture—60 cal yr BP	3,783 (523,884)	463 (10,789)	4,246	51.70	95.82
Salix—Capture—1900 cal yr BP	5,024 (564,634)	571 (11,785)	5,595	55.70	88.35
Salix—Capture—5,400 cal yr BP	1,227 (57,425)	66 (576)	1,293	33.30	55.37
Salix—Capture—6,700 cal yr BP	1,082 (41,608)	82 (898)	1,164	32.40	51.21
Salix—Shotgun—60 cal yr BP	5,647 (6,970)	161 (184)	5,808	75.70	95.06
Salix—Shotgun—1900 cal yr BP	7,289 (8,740)	185 (207)	7,474	77.80	87.66
Salix—Shotgun—5,400 cal yr BP	1,060 (1,117)	15 (16)	1,075	43.40	57.36
Salix—Shotgun—6,700 cal yr BP	545 (574)	10 (11)	555	30.30	47.84

Note

Relative = % of sample reads relative to the corresponding sample reads sum of *Alnus*, *Betula*, and *Salix*.

**FIGURE 10:**
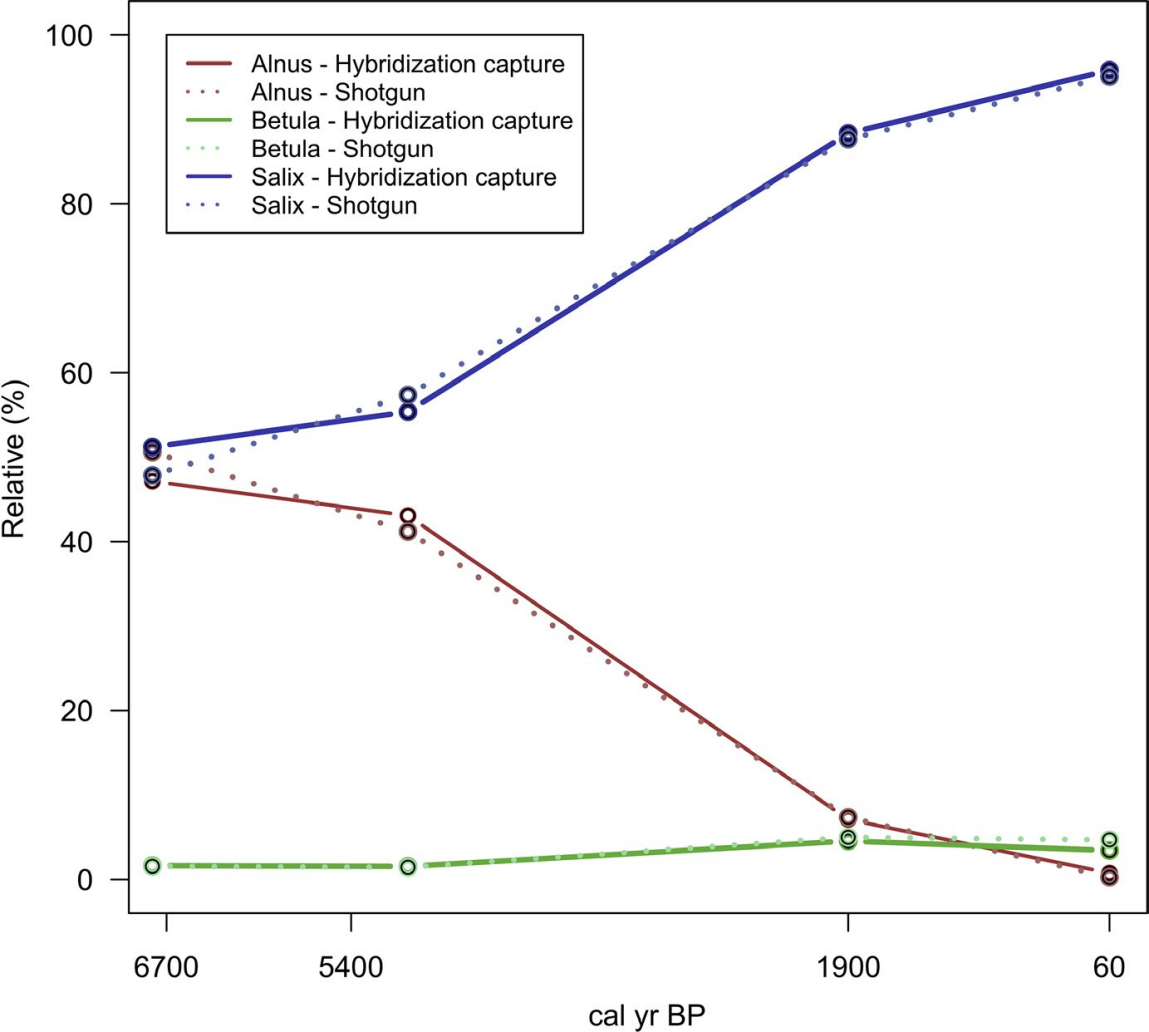
Percentage of sample reads relative to the corresponding sample read sum of *Alnus*, *Betula* and *Salix* in hybridization capture and shotgun dataset

To evaluate the abundance of *Alnus*, *Betula*, and *Salix*, we calculated the percentage of sample reads relative to the corresponding sample read sum of *Alnus*, *Betula*, and *Salix* (Table 3). *Alnus* and *Salix* showed comparable relative percentages of reads at 6,700 and 5,400 cal yr BP (Table 3; Figure 10). An increase of *Salix* and a decrease of *Alnus* reads were visible between 5,400 cal yr BP and 60 cal yr BP. The surface sample (60 cal yr BP) showed the lowest relative percentage of *Alnus* and the highest relative percentage of *Salix*. A slight increase of *Betula* was detected between 5,400 and 1900 cal yr BP.

#### 3.2.2 Comparison of genetic variations between modern individuals and lake sedimentary DNA

Reads assigned to *Alnus*, *Betula,* and *Salix* in the metagenomics shotgun sequencing and hybridization capture experiments were aligned to the corresponding assembled modern chloroplast genomes to evaluate patterns of genetic variations through time. Only the SNP positions previously detected among the assembled modern chloroplast genomes were evaluated.

The results showed that 13 out of 16 variations previously identified in our chloroplast genome sequences from modern *A. alnobetula* assemblies were covered by sedaDNA reads from shotgun metagenomics or hybridization capture experiments (Figure 5). All of the capture and shotgun reads showed the variant only detected in the Taymyr region, and all detected variation were congruent with the potential diagnostic variations for the Taymyr region revealed in modern samples (Figure 5, position highlighted in red), as well as one variation (Figure 5, position highlighted in light green) for Omoloy and one variation for Kolyma (Figure 5, position highlighted in blue).

From the 61 SNPs detected in our *B. nana* assemblies, 44 SNPs were covered by sedaDNA reads from shotgun metagenomics or hybridization capture. Twenty variations match a variant shown by either B01 or B02 individuals from Taymyr (Figure 7, variants highlighted in red), while 24 variations match both B01 and B02 individuals. Since the sedaDNA (CH12 core from Taymyr) data showed variants present only in the individuals from Taymyr or Omoloy, seven out of nine variations (Figure 7, position highlighted in blue) were identified as potential biogeographic markers for Kolyma. The read count and breadth of coverage for *Betula* were very low (1%–2%) in the deeper core samples (6,700 and 5,400 cal yr BP).

For *Salix*, sedaDNA reads covered 31 (97%) of the variations identified between the *Salix* sp. (S01) assembly and the *Salix purpurea* reference chloroplast genome (Figure 8). Fifteen (48%) and five (16%) variations found in capture and shotgun data were in accordance with the individual S01 or the reference *Salix purpurea,* respectively. Whereas, ten variations (32%) showed both the individual and the reference variants.

To further substantiate the reliability of the sediment core data, the complete dataset of sedaDNA reads were aligned to each of the modern genomes. The results reported in Table S4 and Figure S1 show that the number of “identical sites” are consistently higher in the individuals from the Taymyr region in comparison to the individuals from Omoloy and Kolyma regions.

## 4 DISCUSSION

### 4.1 De novo assembly of chloroplast genomes in modern shrub individuals

#### 4.1.1 Genomes and genetic variation

The assembled chloroplast genomes of all *A. alnobetula*, *B. nana*, and *Salix* sp. individuals have the same gene number and gene order as the corresponding reference chloroplast genomes. The size of the generated *A. alnobetula* draft chloroplast genome is 22 bp larger than the reference genome (accession no.: MF136498.1; Gryta et al., 2017) due to an expansion of 29 bp at the IRb/SSC border and a contraction of 7 bp at the LSC/IRb border in all *A. alnobetula* individuals. The IR borders are among the most variable chloroplast coding regions in many plant species, likely under purifying selection (Firetti et al., 2017; Gu et al., 2019; Yang et al., 2018, 2019), and with a putative role in chloroplast genome stability and evolution (Yang et al., 2019; Zhu et al., 2016). The gene *ycf1*, lying at the IRs/SSC boundaries in all our assemblies, shows two SNPs in one *A. alnobetula* individual from Kolyma and three SNPs in the *Salix* sp. individual. Previous studies proposed *ycf1* as a promising cpDNA barcode of land plants due to its species‐level resolution (Dong et al., 2015). The SNPs identified in our study are therefore evaluated as candidate biogeographic markers for *A. alnobetula* individuals from the Kolyma region and *Salix* sp. individuals from Omoloy.

#### 4.1.2 Phylogeography

The genetic variations detected in the assemblies can be used to explore their biogeographical relationships (Wu, 2015; Yang et al., 2018), although due to the small sample size, our results represent a population subset and a partial genetic characterization. Parsimony‐based chloroplast haplotype networks of *A. alnobetula* show a clear geographical structure. The haplotypes within the Omoloy and Kolyma populations are more closely related to each other than the *A*. *alnobetula* lineage from Taymyr. In accordance with previous studies (Eidesen et al., 2013), our result suggests that the Lena River might serve as a barrier to gene flow between the Taymyr region and the two eastern regions, Omoloy and Kolyma. Our results are in line with former studies of *A*. *alnobetula* from other geographical regions (Douda et al., 2014; Gryta et al., 2017; Hantemirova et al., 2018), which all report low intrapopulation variability and pronounced phylogeographic structure in this species. Considering the extensive geographic distances between Taymyr, Omoloy, and Kolyma, we detected a lower intrapopulation variability than studies on smaller areas of Europe and southern Asia (Douda et al., 2014; Gryta et al., 2017; Hantemirova et al., 2018). Overall, we identified 11 SNPs and 2 insertions as potential candidate biogeographic markers for the Taymyr region, 1 SNP for Omoloy, and 1 SNP for Kolyma which can be screened in future studies with larger sample sizes covering more populations.

In *B. nana*, we identified 9 SNPs as potential biogeographic markers for Kolyma, while no specific variant was found for Taymyr or Omoloy. We found higher genetic variation but no clear spatial structure among *B*. *nana* individuals. Although *B. nana* produces smaller amounts of pollen and seeds compared to tree birches (Wang et al., 2014), its seeds are smaller and lighter in comparison to A. *alnobetula*, which might maintain higher genetic variability in *B. nana* chloroplast genomes (Alsos et al., 2012). Previous studies show that the cpDNA haplotypes can be extensively shared among populations of *Betula*, both within and between species (Palmé, 2003), which may confound phylogeographic patterns. However, in our study areas, *B. nana* is not growing in sympatry with their southerly neighboring tree birch species (*B. pubescens* and *B. pendula*) (Andreev et al., 2002; Niemeyer et al., 2015; Safronova & Yurkovsksya, 2019). The study areas are mostly isolated from tree birches by monodominant larch forests. Therefore, we assume that the chance of modern hybridization is negligible. Moreover, previous studies have shown that gene flow into *B. nana* from *B. pubescens* is less likely due to the reproductive barrier between diploids and tetraploids (Zohren et al., 2016). On the other hand, there is evidence that between 8,000 and 9,000 yr BP *B. pendula* spread to 72°N in the Taymyr region, due to warmer summer temperatures (Kremenetski et al., 1998). Hybridization and introgression processes with diploid neighboring species (e.g., *B*. *pendula*) could therefore have happened in the past and be a possible source of haplotype sharing in *Betula* (Da̧browska et al., 2006; Eidesen et al., 2015; Jadwiszczak et al., 2012; Salojärvi et al., 2017; Li et al., 2005; Palmé, 2003; Thomson et al., 2015). Although we did not perform sequence divergence or allele frequency distribution analysis with neighboring birches, our hypothesis might serve as a stimulus for future studies.

Overall, the low genetic diversity among *A*. *alnobetula* from Taymyr, Omoloy, and Kolyma suggests that the recent northward shrubification might be due to local population recruitment and growth response of the already present local community. In contrast, the higher genetic variability in *B*. *nana* suggests that recruitment might originate from different populations due to more efficient seed dispersal, increasing the genetic connectivity over long distances, whereas *A. alnobetula* seeds are larger and heavier and consequently have a shorter dispersal distance. The de novo assembled chloroplast genomes, the potential biogeographic markers, and the chloroplast haplotype networks provided in our study provide a basis for further investigations of past and present shrub population dynamics in northern Siberia and their phylogenetic relationship. Due to small sample size, we used the modern genomes mainly to guide the analyses of ancient genomes rather than having a comprehensive genome characterization of modern individuals.

### 4.2 Past shrub population dynamics at the Siberian tundra‐taiga ecotone

In order to investigate shrub paleodynamics, we used both metagenomic shotgun sequencing and hybridization capture enriched sequencing data retrieved from four lake sedaDNA samples by Schulte et al. (2020). The reads were taxonomically assigned to *Alnus*, *Betula,* and *Salix* genus. The hybridization capture data show a lower breadth of coverage then shotgun data on average due to the specificity of the hybridization capture baits for targeting the chloroplast genome of *L. gmelinii* (Schulte et al., 2020; Zimmermann et al., 2019). Only the cp conserved regions of *Alnus*, *Betula*, and *Salix* were captured, of which only a reduced pool can be uniquely assigned to specific taxa using the lowest common ancestor approach. However, the proportions of read counts and breadth of coverage among the taxa are comparable between the capture and shotgun data. Previous studies have shown the potential of the hybridization capture method to retrieve diverged sequences up to a maximum of 25%–40% in different studies on chloroplast, mitochondrial, and nuclear genomes (Li et al., 2013; Paijmans et al., 2016; Peñalba et al., 2014). Our study reveals the reliability and feasibility of hybridization capture for plant biodiversity studies on ancient DNA through the enrichment of diverged sequences. The capturing of conserved chloroplast regions using a set of diverged targeted species could lead to a detailed understanding of genetic variations as well as a broader picture of biodiversity changes through time.

Our results show a decrease of *Alnus*, a slight increase of *Betula,* and a significant increase of *Salix* in the Taymyr region during the last 5,400 years, reflecting a strong environmental turnover from open *Larix* taiga, mainly characterized by *Alnus* with a low cover of *Betula* and *Salix* sp., to single‐tree tundra (Niemeyer et al., 2017). This confirms previous palynological and metabarcoding investigations of the same sediment core as well as the core from the neighboring lake CH06 which revealed a general larch forest decline along with a decrease of *Alnus* understory during the last ~6,000 years in the Taymyr region (Epp et al., 2018; Klemm et al., 2016). Comparable shrub dynamics were observed by a recent study, using both metabarcoding and pollen data, in two sediment cores at the tundra‐taiga ecotone (70.96–70.53°N) in the Omoloy region. Pollen records from the Nikolay Lake (73°20′N, 124°12′E) reported by Andreev et al. (2004) are as well in line with our results. Therefore, multiple pollen and sedimentary DNA records from lakes which lay at a comparable latitude to our lake CH12 (72.399°N) are in line with our results, revealing similar shrub dynamics along the Siberian tundra‐taiga ecotone. The mid‐Holocene vegetation was characterized by open *Larix* taiga with *Alnus* shrubs in the understory (Klemm et al., 2016; Naito & Cairns, 2011; Zimmermann, Raschke, Epp, Stoof‐Leichsenring, Schirrmeister, et al., 2017). The modern vegetation of shrub tundra, dominated by sedges and grasses with only sparse *Larix* stands, became established at approximately 2,200 cal yr BP (Epp et al., 2018; Klemm et al., 2016). A similar timing of strong change has also been reported from other sites in the Taymyr region (Andreev et al., 2011; Klemm et al., 2016) and throughout most circumarctic environments (Epp et al., 2018; Kaufman et al., 2004; Klemm et al., 2016; Luoto et al., 2014; Salonen et al., 2011). The beginning of the Holocene was characterized by an increase in summer temperature by 5–6°C in Taymyr followed by a climate deterioration and the reduction of the *Alnus fruticosa* range after 5,000 cal yr BP (Kremenetski et al., 1998). *Alnus* may have retreated southwards together with *Larix* while *Salix* spp. and *Betula* spp. replaced *Alnus* spp. in its once favored places. However, possible overrepresentation of *Salix* sp. via both the source signal (i.e., *Salix* growing close to the lake and tributary rivers rims) and preservation signal (i.e., differences in the original DNA content of the leaves and DNA degradation) need to be taken into account (Forbes et al., 2010; Niemeyer et al., 2017).

The hybridization capture and shotgun sequencing reads were aligned to the individuals’ chloroplast genomes of the corresponding taxa in order to compare the genetic variations previously identified among the individuals with the sedaDNA variants. All the genetic variants detected in the *Alnus* individuals from the Taymyr region correspond to the variants in the sedaDNA. Eight potential markers for Taymyr geographic discrimination in *Alnus* are supported by the sedaDNA data. This shows that genetic variants were maintained through the last 6,700 years in *Alnus* individuals from the Taymyr region. Furthermore, the higher “identical sites” between the full sedaDNA dataset and the individuals’ chloroplast genomes from the Taymyr region strengthen the reliability of sedaDNA data. Whereas, sedaDNA from *Betula* shows a pattern of variations in accordance with the higher genetic diversity observed in modern individuals: 18% and 29% of variations match with Taymyr individuals B01 and B02, respectively, while 54% of variants match with both. Seven variations are confirmed as potential markers for Kolyma geographic discrimination of *Betula* since they were not present in sedaDNA.

Our study shows the hybridization capture method potential to retrieve specific genetic variants in sedaDNA. Future method development toward specific targets’ enrichment could lead to a detailed understanding of ancient genetic variations. Future studies investigating shrub genetic diversity and population dynamics through time using the haplotype and the potential markers identified in our study can now be undertaken. This would allow a better understanding of past shrub dynamics due to glacial contractions and postglacial population expansions.

## 5 CONCLUSIONS

Current environmental changes, particularly climate warming and its feedback mechanisms are affecting plant composition and biomass of arctic and alpine ecosystems. The increase in relative abundance and cover of deciduous shrubs in sub‐arctic Siberian areas is rather well documented (Frost & Epstein, 1960s). To investigate the dynamics of shrubification, we sequenced and assembled 15 chloroplast genomes based on modern DNA (7 *A*. *alnobetula*, 7 *B*. *nana,* 1 *Salix* sp. individual). We identified genetic variations and estimated haplotype networks for the chloroplast genomes of each taxon. To infer prehistorical genetic variation and past population dynamics, we used taxonomically assigned metagenomics shotgun and hybridization capture sequencing data retrieved from four sedaDNA samples by Schulte et al. (2020). The samples were obtained from four different age segments of a sediment core from lake CH12 in the Taymyr region. Genetic variations identified in modern individuals were then compared with sedaDNA variants.

The low genetic diversity and the spatial structure of haplotypes among modern *A*. *alnobetula* individuals suggest that the recent northward shrubification might be due to local population recruitment and growth response of already present local communities, whereas the higher genetic variability and absence of a spatial signal in *B*. *nana* might indicate recruitment from different populations due to more efficient seed dispersal. The analysis of sedaDNA reveals a decrease of *Alnus* and an increase of *Betula* and *Salix* in the Taymyr region over the last 5,400 years, reflecting the strong environmental turnover from open *Larix* taiga to shrub tundra. The comparison of modern DNA with sedaDNA shows that genetic variants were maintained throughout the last 6,700 years in *Alnus* individuals from the Taymyr region, while *Betula* and *Salix* have higher genetic diversity in both modern individuals and sedaDNA. The success of the hybridization capture in retrieving diverged sequences demonstrates the high potential for future studies of plant biodiversity as well as specific genetic variation on ancient DNA from lake sediments. Overall, our study provides new insights into past and modern genetic signatures as well as shrub vegetation dynamics through time at the Siberian tundra‐taiga ecotone. Further research is needed to gain a better understanding of past population dynamics in relation to the current shrubification which has probably already begun to alter ecosystem properties.

## CONFLICT OF INTEREST

The authors declare no potential conflicts of interest.

## AUTHOR CONTRIBUTION

**Stefano Meucci:** Conceptualization (equal); Data curation (equal); Formal analysis (equal); Investigation (equal); Methodology (equal); Visualization (equal); Writing‐original draft (equal). **Luise Schulte:** Data curation (equal); Formal analysis (equal); Investigation (equal); Methodology (equal); Resources (equal); Software (equal); Supervision (equal); Writing‐review & editing (equal). **Heike Zimmermann:** Conceptualization (equal); Data curation (equal); Formal analysis (equal); Investigation (equal); Methodology (equal); Resources (equal); Software (equal); Supervision (equal); Writing‐review & editing (equal). **Kathleen Stoof‐Leichsenring:** Resources (equal); Writing‐review & editing (equal). **Laura Epp:** Writing‐review & editing (equal). **Pernille Bronken Eidesen:** Writing‐review & editing (equal). **Ulrike Herzschuh:** Conceptualization (equal); Funding acquisition (equal); Project administration (equal); Resources (equal); Supervision (equal); Writing‐review & editing (equal).

## Supporting information

Fig S1

Table S1

Table S2

Table S3

Table S4

Supplementary Material

## Data Availability

Chloroplast genome assemblies: The samples have been submitted to NCBI under the GenBank accession numbers available in Table 1 and under the BioProject PRJNA616329. Sedimentary ancient DNA sequenced by hybridization capture and shotgun sequencing methods: The Illumina sequence data are submitted to the European Nucleotide Archive under project number PRJEB35838, accession numbers ERS4197088 ‐ ERS4197099.
