# Supplementary figures and images for: Holocene chloroplast genetic variation of shrubs (Alnus alnobetula, Betula nana, Salix sp.) at the siberian tundra‐taiga ecotone inferred from modern chloroplast genome assembly and sedimentary ancient DNA analyses
Source: Ecol Evol. 2021 Jan 31;11(5):2173–93. doi: 10.1002/ece3.7183 (PMC7920767; doi:10.1002/ece3.7183)

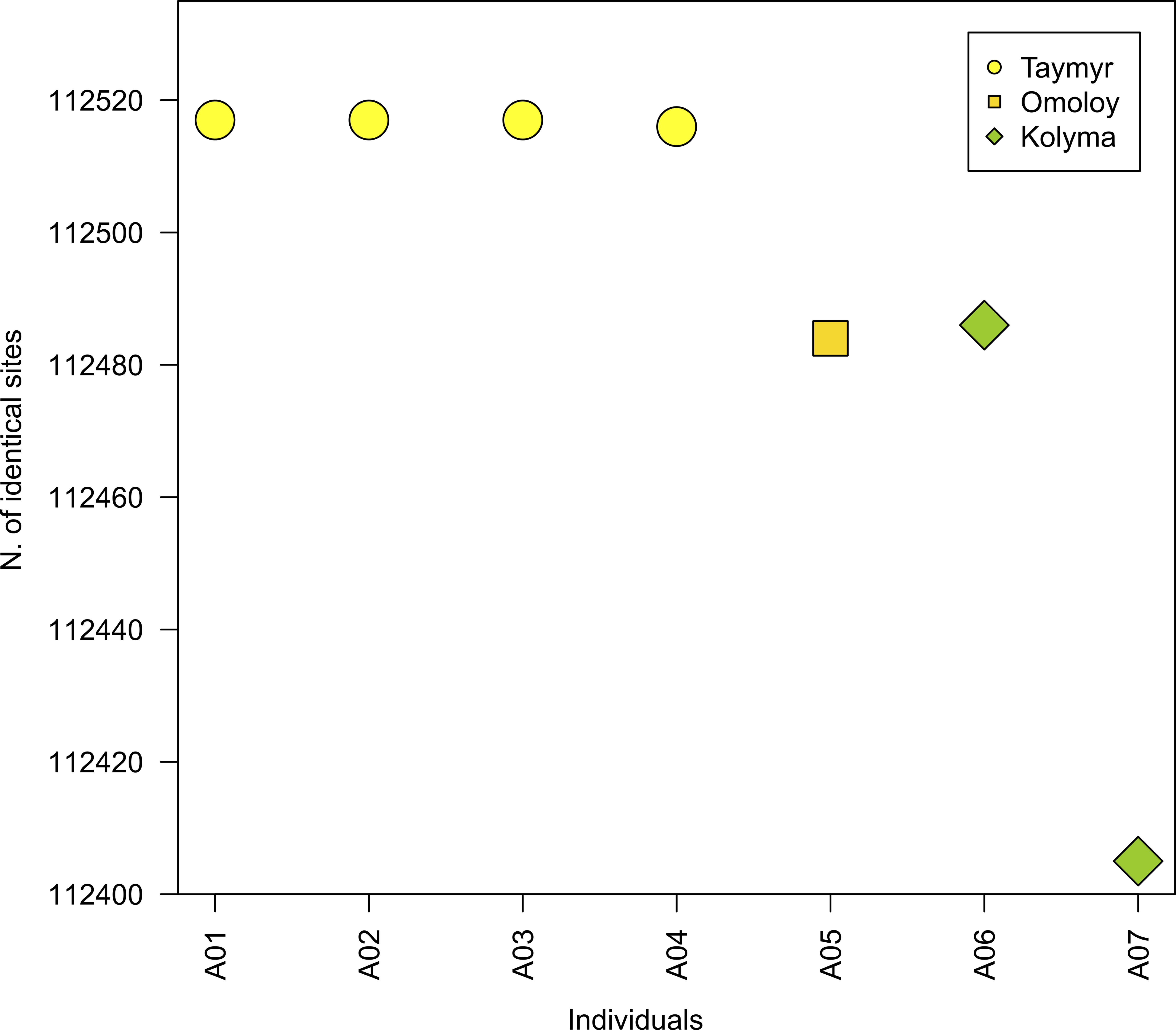

Supplement: Supplementary file 1 — Fig S1 [file ECE3-11-2173-s001.jpg]
