# Supplementary material for: Holocene chloroplast genetic variation of shrubs (Alnus alnobetula, Betula nana, Salix sp.) at the siberian tundra‐taiga ecotone inferred from modern chloroplast genome assembly and sedimentary ancient DNA analyses
Source: Ecol Evol. 2021 Jan 31;11(5):2173–93. doi: 10.1002/ece3.7183 (PMC7920767; doi:10.1002/ece3.7183)
Supplement: Supplementary file 6 — Supplementary Material [file ECE3-11-2173-s006.docx]

**Supplementary Material**

**Supplementary Figure 1.** Number of identical sites between the full capture and shotgun dataset with each *Alnus* individuals’ chloroplast genomes.

**Supplementary Table S1.** Chloroplast genome assemblies: Mapping reads, depth of coverage, total chloroplast genome length, IR length

**Supplementary Table S2:** Insertion–deletion mutations (InDels) detected in each of the whole chloroplast gnome alignments of the 7 *A. alnobetula,* 7 *B. nana* and *Salix* sp. individuals. The reference chloroplast genome and the sedaDNA retrieved from core samples through hybridization capture and shotgun sequencing methods were mapped subsequently in order to evaluate their variants corresponding to the InDels’ positions. An InDel’s position corresponds to the reference chloroplast genome. If an InDel is located within a gene, the corresponding gene name is given in the first row. If no reads were retrieved from core samples, no variant is reported.

**Supplementary Table S3:** Taxonomic assignment of reads to *Alnus*, *Betula,* and *Salix* for each capture and shotgun sample, performed with Kraken 2 (Materials and methods). Abbreviations: Relative = % of sample reads relative to the corresponding sample reads sum of *Alnus*, *Betula,* and *Salix*.

**Supplementary Table S4:** Alignment statistics between the full capture and shotgun dataset with each *Alnus* individuals’ chloroplast genomes.
